# A Poly-L-Lactic Acid Honeycomb Scaffold for Enhanced Cytocompatibility

**DOI:** 10.3390/polym18212800

**Published:** 2026-11-09

**Authors:** Petr Slepička, Iva Labíková, Bára Frýdlová, Matěj Budina, Petr Sajdl, Václav Švorčík, Nikola Slepičková Kasálková

**Affiliations:** 1 Department of Solid State Engineering, University of Chemistry and Technology Prague, 166 28 Prague, Czech Republic; 2 Department of Power Engineering, University of Chemistry and Technology Prague, 166 28 Prague, Czech Republic

**Keywords:** biopolymer, poly-L-lactic acid, honeycomb pattern, plasma treatment, surface morphology, cytocompatibility

## Abstract

In this study, we present the development of biopolymer honeycomb (HCP PLLA) microstructures on plasma-modified PTFE polymer using the improved phase separation method with the aim of preparing a surface with enhanced cytocompatibility. To determine changes in surface morphology and characterize the size and shape of the prepared PLLA microstructures, laser confocal microscopy (LCM), atomic force microscopy (AFM), and scanning electron microscopy (SEM) methods were used. To determine the chemical composition of the surface of substrates modified with the formed HCP PLLA microstructures, X-ray photoelectron spectroscopy (XPS) was used. PTFE samples modified with an 8 W plasma discharge proved to be a suitable material for further study as a substrate for honeycomb patterns and biocompatibility testing. It was found that the formation (size) of PLLA microstructures is influenced not only by the exposure time of the samples in plasma but also by the PLLA content in the coating solution and the thermal stress of the material. Plasma modification and subsequent phase separation significantly increased the surface amount of oxygen and carbon and suppress the surface amount of fluorine to a minimum, which is evidence of almost 100% coverage of the PTFE surface with the PLLA coating solution. Biocompatibility testing on selected PTFE samples revealed that modification with 8 W plasma in combination with HCP microstructures—created by the phase separation method in solution with 3 wt. % PLLA—leads to significant support for SaOS-2 osteoblast cell proliferation. This study highlights the potential of biopolymer honeycomb pattern surfaces with engineered topography for advanced biomedical applications.

## 1. Introduction

Highly ordered two-dimensional microporous materials with hexagonal structures have attracted considerable interest in many areas of materials science and nanobiotechnology, such as organic optoelectronics [1,2,3], advanced electronics [4,5], tissue engineering [6,7], membrane preparation [8,9] and microtemplates [10,11,12]. Over the past few decades, a number of so-called “top-down” and “bottom-up” techniques have been developed, including lithography, phase separation of block copolymers, colloidal templates, the Breath Figure method, etc. These methods can prepare highly ordered structures with uniform pore sizes on the nano- and micrometer scales [13]. It has been found that the surface properties of two-dimensional monolayers or three-dimensional multilayers on polymer scaffolds play a crucial role in cell cultivation—i.e., in surface adhesion, migration, proliferation and differentiation [6,14]. Furthermore, it has been shown that cell proliferation and surface adhesion on microporous films with hexagonal microstructures on the surface are influenced by pore size [15]; moreover, cell signalling is more effectively activated on microporous polymer films prepared in this way than on smooth surface films [6,7]. The Breath Figure technique is widely used in a number of applications due to its speed of preparation, relative simplicity, low cost, and the possibility of uniform structuring of the entire substrate surface [16]. The principle of this method is based on the application of a polymer solution in a non-polar solvent to a substrate that rotates in air with a relative humidity of more than 50%. Evaporation of the solvent and cooling of the air just above the polymer cause condensation of water droplets on a polymer layer. The condensed droplets form a hexagonal system and are transported to the substrate surface by capillary forces. If external parameters such as temperature and air humidity and process parameters (e.g., polymer concentration, solvent, etc.) are optimally set, highly organized porous surfaces can be prepared using this technique. By controlling these parameters, pore size can be influenced. The name of this technique is derived from the fact that the formation of condensed droplets on the substrate surface occurs due to the same principle as breathing on a cold surface [17,18]. As described in their study by Kim et al. [19], the formation of monolayer or multilayer porous films is fundamentally influenced by the density of the solvent used: using solvents with a density greater than water (e.g., CS_2_) produces multilayer structures, while using solvents with a density less than water (e.g., benzene, toluene) produces monolayer structures [17].

Although the Breath Figure method for the preparation of highly ordered porous films has many of the above-mentioned advantages, there are also a few shortcomings that are critical obstacles to the industrial expansion of this method and its application potential. These include the requirement for very humid ambient conditions and low applicability to commercial polymers. Several attempts have been made to prepare HCP-shaped porous structures in a dry environment by imitating the ambient humid atmosphere by introducing water or low-volatile poor solvents into the polymer solution [19,20,21,22]. However, the uniformity and orderliness of the formed porous patterns were relatively low due to the difficulty of stabilizing the droplets from the prepared emulsion. For this reason, other methods are being developed for the mass production of porous films with highly ordered structures (honeycomb structures) under normal ambient conditions without the need for high air humidity and with applicability to commercially available polymer materials [23]. Bui et al. [23] in their study present a two-step technique, which is referred to as the “improved phase separation” (IPS) method. Thanks to this method, porous films with HCP structures can be prepared on a large scale under normal air conditions. In an attempt to simplify the preparation process while maintaining the advantages of this method, Bui et al. [23] developed a new one-step technique for preparing structures on the surface of polymer films by further modifying the IPS method. In this approach, a certain amount of methanol is introduced directly into the polymer solution in chloroform, thereby creating a ternary system of polymer–“good solvent”–“bad solvent”. By immersing the substrate in the as-prepared solution or by casting the solution onto the surface of a solid substrate and then drying in air with simultaneous evaporation of the solvents, hexagonal ordered honeycomb structures are formed. This technique can be applied to a number of commonly available commercial polymers, including, e.g., biocompatible and biodegradable poly(L-lactic acid) [24,25,26], polystyrene [27,28], cellulose acetate [29,30], or PMMA [31] (the structures which were prepared by our group on plasma treated FEP) [32]. Methanol is highly soluble in water-immiscible solvents such as chloroform, dichloromethane, or carbon disulphide; these solvents are compatible with the formation of ordered honeycomb structures [33].

Structured porous surfaces, whether at the micro- or nanoscale, are widely used in the fields of tissue engineering [6,7,17] and organic optoelectronics [2,3,34]; for the production of biosensors [35,36]; and in membranes for shape- and size-selective separation [8,9] as carriers for cell cultures. In the field of tissue engineering and transplantation medicine [37], great emphasis is currently placed on the development of cell scaffolds [37]. In recent years, synthetic polymers have become very important materials. The main idea in the development of synthetic scaffolds is to ensure that cell carriers function as a natural extracellular matrix (ECM). In order to create an advanced biosynthetic tissue or organ after the application of a cell scaffold into an organism, the cell carrier should have the ability to actively stimulate specific cellular responses, instead of just passively tolerating them by the cells [37]. For this reason, cell adhesion is ensured by binding interactions between cell adhesion receptors (so-called integrins) and active parts of ECM molecules or specific amino acid sequences [38]. Bui et al. focused on the preparation of highly ordered porous films with honeycomb structures using the IPS method and found that structured polymers prepared in this way provide a three-dimensional environment in which cells behave the same as in vivo; therefore, these porous films serve as suitable scaffolds for supporting cell adhesion, maintaining differentiation and proliferation [23]. Porous membranes, which are most often prepared using the breath figure method, can be used in a number of other applications, e.g., for the separation of water from oil [39] or EMI shielding [40] (where, in general, the dimensions and homogeneity are of great importance for particular applications) [41].

Although honeycomb-patterned polymer surfaces have been investigated extensively, the formation and biological evaluation of PLLA honeycomb microstructures on PTFE remain insufficiently explored. PTFE is attractive for biomedical applications because of its chemical stability and biological inertness; however, its low surface energy limits coating adhesion and cellular interactions. The present study addresses this limitation by combining plasma activation of PTFE with the deposition of biocompatible PLLA honeycomb structures. This approach integrates the favourable physicochemical properties of PTFE with the topographical and biological advantages of a biodegradable polymer coating. A particularly important contribution is the identification of 8 W plasma treatment as an effective pre-treatment for the subsequent formation of stable PLLA microstructures. Plasma activation improves the compatibility between the highly fluorinated PTFE substrate and the PLLA coating. The improved phase separation method additionally represents a relatively simple and versatile strategy for producing well-defined microstructured coatings. The results demonstrate that honeycomb morphology can be controlled through several processing parameters, including plasma exposure time, PLLA concentration, and thermal treatment. This finding is important because it enables the surface architecture to be tailored for specific biological applications. Overall, this study provides a novel route for transforming biologically passive PTFE into a cytocompatible microstructured platform with potential applications in implant coatings, cell carriers, tissue engineering substrates, and other biomedical devices.

## 2. Materials and Methods

### 2.1. Materials and Substrate Exposure

PTFE foils from Goodfellow Ltd (Huntingdon, UK). with a thickness of 50 μm and a density of 2.2 g.cm^−3^ were used for the experiment. To modify the physicochemical properties of PTFE substrates using plasma modification with argon plasma, a desktop sputter coater, model SCD 050 from BAL-TEC, was used in etching mode. PTFE samples were plasma-modified for 20 s and 240 s, and the discharge power was set to 3 W and 8 W. Half of the samples (only plasma-treated substrates) were thermally stressed; PTFE samples were heated for 1 h at 300 °C.

### 2.2. Phase Separation Technique

The improved phase separation (IPS) method was used to form HCP structures on plasma-modified and unmodified PTFE. A mixture of two solvents—chloroform (density 1.48 g.cm^−3^, Mr = 119.38 g/mol) and methanol (density 0.791 g.cm^−3^, M_r_ = 32.04 g/mol)—and 50 μm thick, 1.25 g.cm^−3^-density PLLA (L-PLA, biodegradable, transparent, biaxially oriented, with 6% elongation at break; tensile modulus of 3.5 GPa; izod. impact strength of 16 J m^−1^; and tensile strength of 53 MPa) film from Goodfellow Ltd. was used to prepare the solution for thin film preparation. The solvent ratio was set to 90/10, and the amount of PLLA was weighed so that the resulting chloroform/methanol solution contained 3 wt.% PLLA and 2 wt.% PLLA. The PLLA solution was prepared in a fume hood under laboratory conditions by mixing 90 mL of chloroform (CHCl_3_) with 10 mL of methanol (CH_3_OH) and then adding a weighed amount of PLLA. In order to completely dissolve the PLLA in the solvent mixture, the solution was left on a laboratory mixer for 24 h. After the PLLA was completely dissolved in the solvent mixture, plasma-modified and unmodified samples of PTFE were coated from the prepared solution by immersing the substrates in the PLLA solution for 10 s and then leaving them on Petri dishes or coverslips in the air. After the solvents had completely evaporated, the samples prepared in this way were subjected to appropriate analytical methods.

### 2.3. Analytical Methods

#### 2.3.1. Atomic Force Microscopy

The surface morphology and roughness of the pristine and treated films were examined by atomic force microscopy (AFM) using Dimension ICON (Bruker Corp., Billerica, MA, USA). The samples were analysed in Scan-Assyst^®^ mode using a nitride lever, SCANASYST-AIR, with a Si tip (and a spring constant of 0.4 N·m^−1^). We used five independent areas for mean R_a_ calculation for each sample; the standard deviation values did not exceed 5%. NanoScope Analysis software, (version 1.80) was applied for data processing. Surface roughness (*R_a_*) represents the arithmetic mean of the absolute values of height deviations measured from the central plane.

#### 2.3.2. Scanning Electron Microscopy

The morphology of the sample surfaces was also characterized by scanning electron microscopy with FIB-SEM LYRA3 GMU (Tescan Brno, Czech Republic). The acceleration voltage was set to 5 kV. The elemental composition was measured by energy-dispersive X-ray spectroscopy (EDS, analyser X-ManN, 20 mm^2^ SDD detector, Oxford Instruments, Buckinghamshire, UK), while the accelerating voltage for SEM-EDS analysis was set to 10 kV.

#### 2.3.3. Laser Confocal Microscopy

Laser confocal microscopy (LCM) is an optical imaging technique that is able to achieve 3D surface imaging with point scanning of the laser beam and the confocal principle. The software composition of the optical sections then creates height images. The advantage of confocal microscopy is the possibility of placing samples directly on the microscope stage, without using a vacuum chamber (unlike AFM or SEM). The sample is observed in real time, and there is no need for the so-called water-proofing of the substrate surface. An Olympus LEXT OLS 3100 confocal microscope was used to measure the surface morphology of PTFE samples with prepared HCP structures.

#### 2.3.4. X-Ray Photoelectron Spectrometry

The quantitative composition of the surface of PTFE polymer substrates after plasma modification and PLLA layer deposition was obtained using X-Ray photoelectron spectroscopy (XPS). The measurement was performed using an ESCAProbeP spectrometer manufactured by Omicron Nanotechnology Ltd. The analysed sample area was 2 × 3 mm^2^. The sample surface was excited by X-ray radiation from a monochromatic source with an energy of 1486.7 eV. The C 1s, O 1s and F 1s spectra were recorded. The relative abundance of elements was evaluated from the intensity of the peaks in the XPS spectra using the CasaXPS software (version 2.3.17PR1.1).

#### 2.3.5. Wettability

The wettability of the studied samples was determined by measurement of contact angles (CA, θ) using goniometer Advex Instruments (Brno, Czech Republic) connected to the SEE System 8.1 program. Analysis of CA was performed at room temperature with 8 µL drops of distilled water (dyed with methyl violet) and glycerol using a Transferpette^®^ automatic pipette (Brand, Wertheim, Germany) at 6 different positions of 3 samples in parallel and perpendicular directions. Subsequently, the drops were photographed and evaluated by 3 marked points.

### 2.4. Cytocompatibility

The interaction of the polymer surface with tissue cultures was studied by the in vitro method. SaOS-2 cells (human osteoblasts) were cultured on the samples. Substances used in biocompatibility tests were as follows: Dulbecco’s Modified Eagle Medium (DMEM), a medium for mammalian cell culture (Sigma Aldrich, St. Louis, MO, USA); fetal bovine serum (FBS), a serum containing growth factors and low levels of antibodies added to DMEM during cell culture (Sebaf GmbH, Großmehring, Germany); phosphate-buffered saline (PBS), a buffered physiological solution containing 0.15 M NaCl used for rinsing cells; and Texas Red C2-maleimide, a fluorescent dye for staining cell plasma membranes red (Thermo Fisher Scientific, Waltham, MA, USA), which was used for staining SaOS-2 cells. Höchst 33258 was used as a fluorescent dye for staining nuclear chromosomes blue (Sigma Aldrich, St. Louis, MO, USA), applied to SaOS-2 cells. Before all biological tests, the samples were sterilized for 1 h in 75% ethanol. Then, the samples were transferred to culture plates. Some 17,000 cells/cm^−2^ were seeded on each sample. DMEM medium (Sigma, USA) supplemented with 10% fetal bovine serum (Sebaf GmbH, Germany) was used for cultivation. Cultivation was carried out for 24, 96, and 168 h at 37 °C, 85% humidity, and in an air atmosphere with 5% CO_2_.

The field-of-view method was used for evaluating biocompatibility. The method was used to determine the adhesion and proliferation of SaOS-2 cells. It proceeds by determining the number of cells that are fixed and stained directly on the tested substrate from photographs taken using a microscope. After the specified time for cultivation, the samples with cells were rinsed with phosphate-buffered saline (PBS) and fixed with ice-cold 75% ethanol (−20 °C) for 45 min. They were then rinsed again with PBS and stained with a mixture of fluorescent dyes (Texas Red C2-maleimide and Hoechst) for 1 h. Using an Olympus IX51 fluorescence microscope with an Olympus DP 70 digital camera, four independently prepared substrates per modification condition were tested, and 20 photographs of each sample were taken. The number of adhered or proliferated cells was determined by counting cells from photographs using NIS Elements software (vers. 6.10). From the photographs taken, it was possible to determine not only the number of cultured cells but also their homogeneous distribution over the sample surface, the extent of their spread, the area of the cells, and their size and shape. A one-way analysis of variance (ANOVA) followed by Tukey’s honest significant difference (HSD) test was used for data modelling of multiple comparisons. All tests were performed with a confidence interval of 0.95.

## 3. Results and Discussion

### 3.1. Scanning Electron Microscopy and Energy-Dispersive Analysis

Surface modification with argon plasma was performed on PTFE with plasma exposure time and its power as modification variables. The samples were exposed to argon plasma for 20 s and 240 s, and the plasma power was set at 3 W and 8 W. Half of the plasma-treated samples were then thermally stressed in a laboratory hot-air dryer, the Binder ED 400. For comparison, PTFE samples that were not plasma-modified were also exposed to thermal stress. Detailed properties of plasma-modified PTFE have already been presented [41]. Plasma-modified samples, heat-treated and non-heat-treated samples, and the original samples were subsequently subjected to the phase separation method by immersion in a polymer solution containing 2 wt. % and 3 wt.% PLLA dissolved in a mixture of chloroform and methanol in order to create regular HCP structures on the surface of PTFE samples.

**Figure 1:**
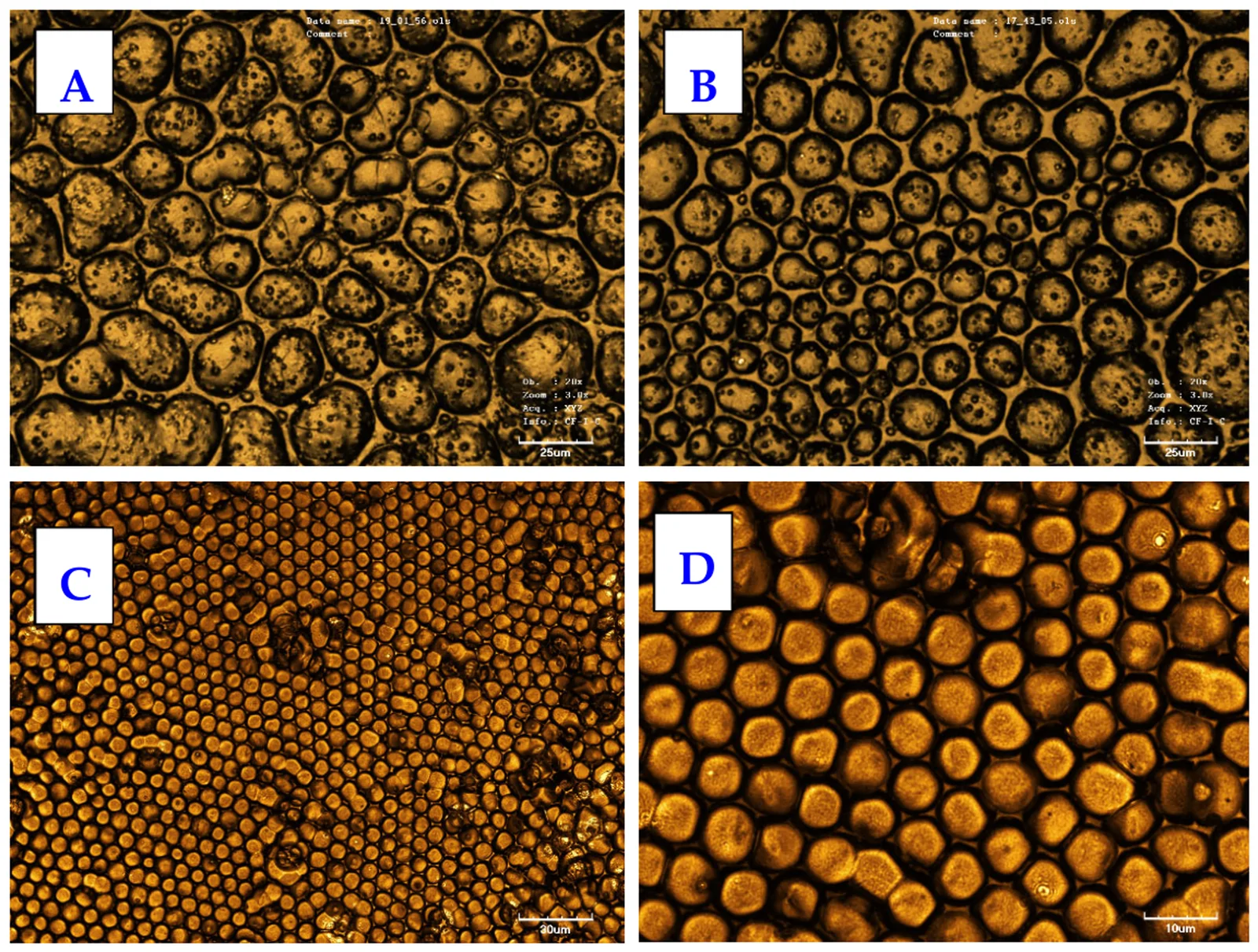
LCM scans of PTFE substrates with created HCP PLLA microstructures. Scans of foils with PLLA content in chloroform and methanol solution 3 wt.%, prepared on (**A**) pristine PTFE; (**B**) PTFE exposed to plasma power of 8 W with a modification time of 240 s and subsequently heat-treated prior to immersion at 300 °C for 1 h; (**C**,**D**) PTFE exposed to plasma power of 8 W with a modification time of 240 s at different magnifications.

First of all, we emphasize that the differences in the final patterns related to heat treatment that we discuss herein will be attributed only to thermal treatment of plasma-exposed PTFE substrate. This heat exposure was selected to “accelerate” the aging process after the plasma exposure and may have strongly contributed to changes in surface chemistry and morphology during the process. From the results of laser confocal microscopy performed after the phase separation method intended for the formation of HCP structures (Figure 1), the PTFE substrate modified with argon plasma with a power of 8 W emerged as the most suitable candidate for biocompatibility testing (causing optimal formation of the pattern); therefore, most of the time-consuming analytical methods intended for the analysis of the resulting microstructures (SEM, AFM) and the chemical composition of the substrate surface (XPS) were performed only on selected samples. PTFE substrates modified with argon plasma with a power of 3 W after phase separation and LCM analysis proved to be a less suitable material for biocompatibility testing due to the resulting surface microstructures (lack of homogeneity); therefore, these samples were subjected only to basic analytical methods (goniometry).

The dependence of the surface morphology of polymer substrates with formed HCP structures on the selected plasma power, plasma exposure time, thermal stress, and PLLA content in the polymer solution was studied using laser confocal microscopy (LCM). Changes in surface morphology depending on air humidity and ambient temperature during the formation of HCP structures by the phase separation method were not taken into account in the evaluation, since these process parameters did not change purposefully during the experiment. Depending on the results obtained during LCM analysis, suitable samples were selected and further studied using atomic force microscopy (AFM) and scanning electron microscopy (SEM). Thanks to these analytical methods, information was obtained about the size and shape of the formed HCP structures on PTFE substrates in the form of 2D and 3D images. The following table (Table 1) summarizes the results obtained during the analysis of HCP structures on PTFE polymer substrates subjected to plasma modification with an argon plasma power of 8 W, modification time of 20 s and 240 s, thermal stress (300 °C, 1 h), and subsequent phase separation from a polymer solution in a mixture of chloroform and methanol with a PLLA content of 2 wt.% and 3 wt.%.

Based on the shape and size of the HCP structures, six samples of PTFE modified with argon plasma with a power of 8 W were selected for further analysis. PTFE substrates modified with a plasma power of 3 W also did not prove to be a material suitable for further analysis, because the resulting microstructures did not correspond in shape to classic HCP structures. Their size was not sufficient compared to the structures formed on PTFE substrates after modification with a plasma power of 8 W, and there was no uniform coverage of the PLLA layer over the entire surface of the substrates.

### 3.2. Atomic Force Microscopy

It can be concluded that the formation of HCP structures on polymer substrates, their final shape, and their size are greatly influenced not only by the plasma power during plasma modification but also by the modification time (Figure 1), the amount of PLLA in the polymer solution, and, last but not least, the effect of thermal stress of the substrate.

The “optimal” coverage of the PTFE sample showed HCP structures evenly covering the entire surface of the sample and proved to be the best candidate in biocompatibility tests (Figure 1C,D). Selected PTFE samples with formed HCP structures and their surface morphology were further analytically studied using AFM and SEM methods.

**Figure 2:**
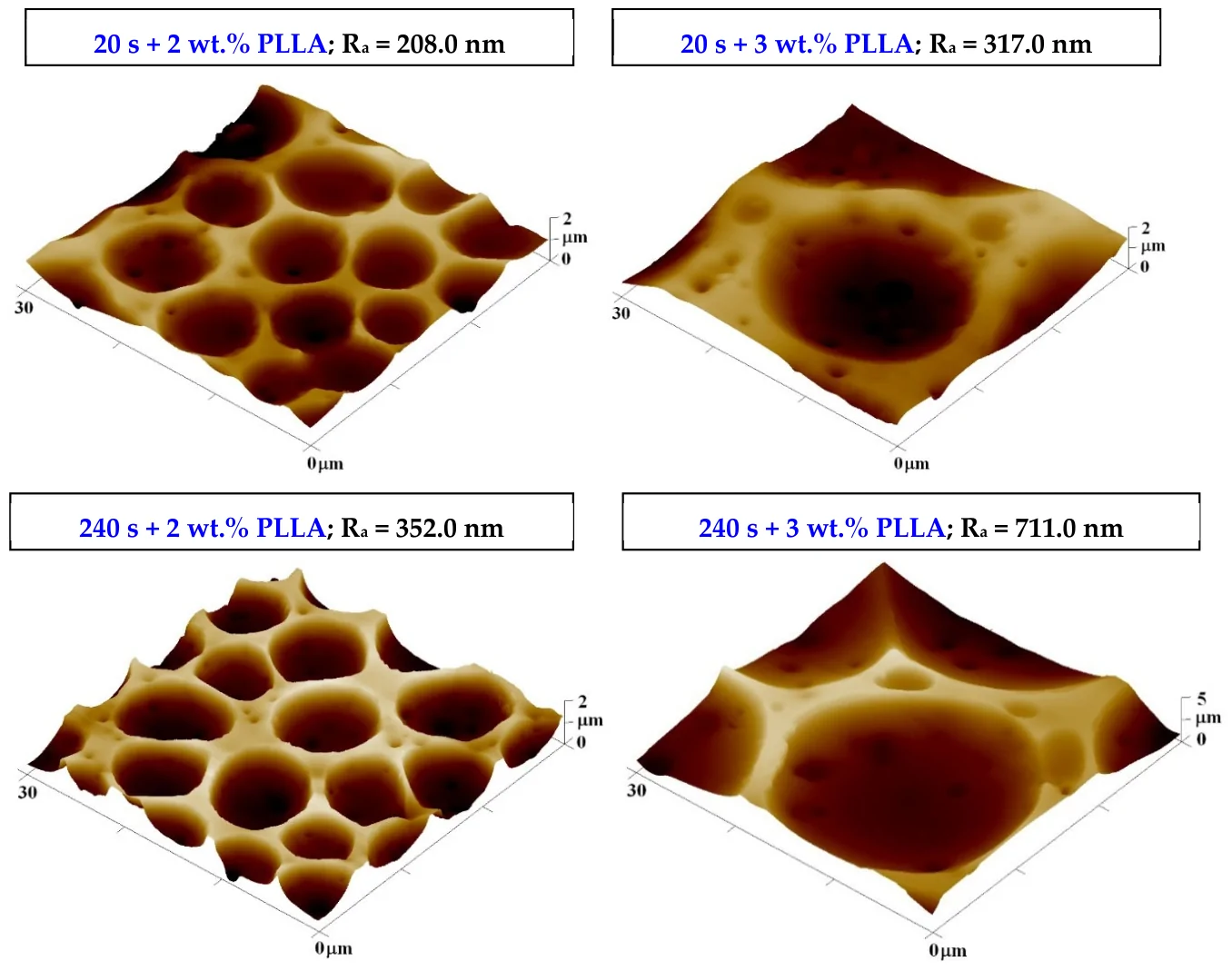
Three-dimensional AFM scans of PLLA on PTFE samples showing the dependence of the size of HCP structures on the PLLA content in the CHCl_3_/CH_3_OH solution (plasma power 8 W, exposure time of 20 s or 240 s, no heat treatment, and PLLA concentration of 2 wt. % and 3 wt.%). The particular description of each sample is presented above each one; the number represents the average surface roughness.

**Figure 3:**
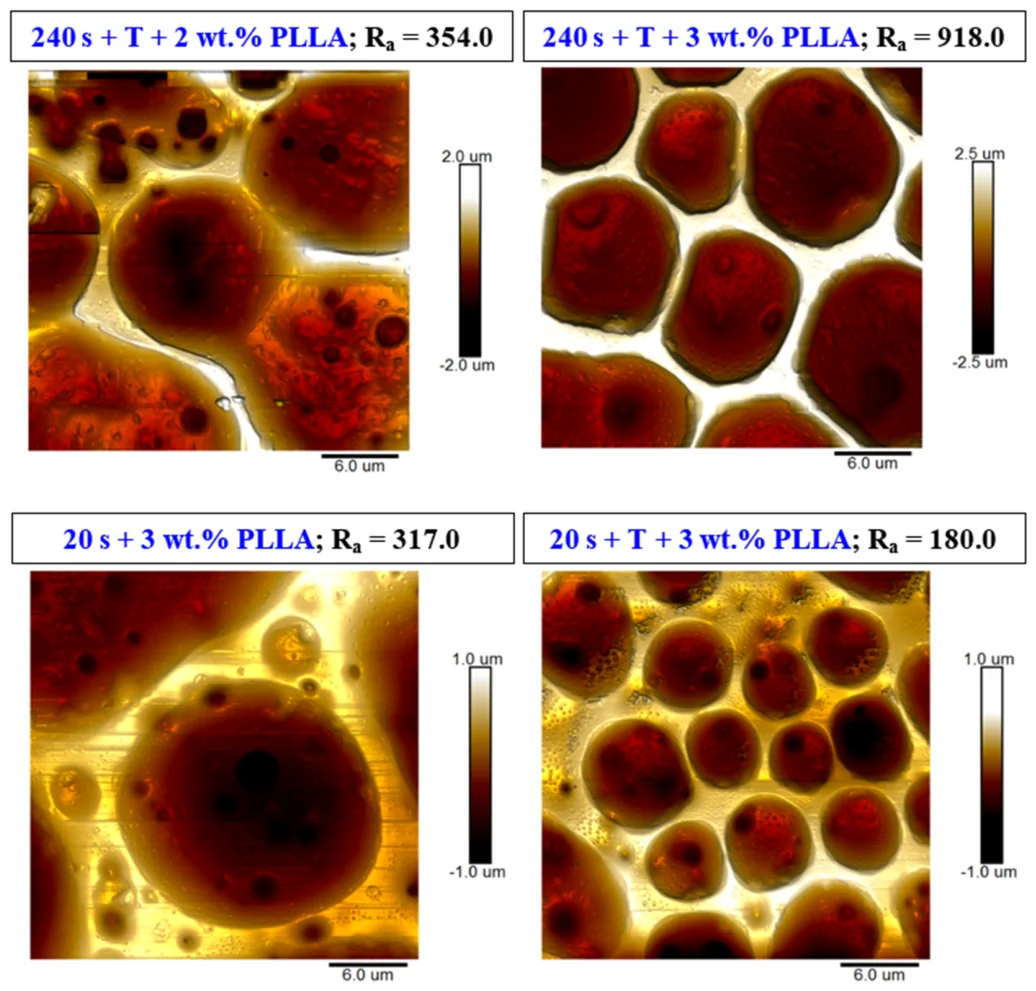
Two-dimensional AFM scans of PTFE samples showing the dependence of the size of HCP structures on the PLLA content in the CHCl_3_/CH_3_OH solution (plasma power 8 W, exposure time of 20 s and 240 s, without/with heat treatment, and PLLA concentration of 2 wt.% and 3 wt.%). The particular description of each sample is presented above each one; the number represents the average surface roughness.

The AFM images in Figure 2 and Figure 3 demonstrate that the morphology of the PLLA honeycomb-patterned (HCP) layer formed on plasma-treated PTFE is strongly affected by the PLLA concentration, duration of plasma exposure, and subsequent thermal treatment. All investigated samples exhibit distinct surface depressions surrounded by elevated polymer walls, confirming the successful formation of porous HCP structures. For the detailed AFM scans (right column, 10-micron scans), the larger honeycomb units formed over the surface were chosen. Nevertheless, substantial differences were observed in pore dimensions, spatial organization, vertical relief, and average surface roughness (R_a_). For the non-thermally treated samples shown in Figure 2, the combination of a lower PLLA concentration of 2 wt.% and a short plasma exposure of 20 s produced relatively small, approximately circular pores distributed over the entire scanned area. Although some variation in pore diameter was evident, the structure remained comparatively homogeneous, with an R_a_ value of 208 nm. Increasing the PLLA concentration to 3 wt.% under the same plasma conditions led to a considerably coarser morphology dominated by a large central depression and several smaller surrounding cavities. The corresponding roughness increased to 317 nm. This development suggests that the more concentrated solution produces a thicker and more viscous polymer layer, in which adjacent phase-separated domains can grow or coalesce before complete solidification.

Prolonging the plasma treatment from 20 to 240 s had an additional pronounced effect. At 2 wt.% PLLA, the longer treatment produced a relatively well-developed, closely packed honeycomb morphology with clearly defined pores and elevated boundaries. Compared with the corresponding sample treated for 20 s, the surface roughness increased from 208 to 352 nm. The more homogeneous distribution and clearer separation of the individual pores may indicate improved spreading and stabilization of the PLLA solution on the more extensively activated PTFE surface. Prolonged plasma exposure can increase the surface energy and wettability of PTFE, thereby altering the interfacial interactions involved in phase separation and polymer deposition. However, at a PLLA concentration of 3 wt.%, the 240 s plasma treatment resulted in a much coarser structure characterized by a large depression surrounded by strongly elevated ridges. The vertical range reached approximately 5 µm, and R_a_ rose to 711 nm. Thus, increasing the polymer concentration from 2 to 3 wt.% approximately doubled the roughness after the longer plasma treatment. These results indicate that the combined effect of stronger substrate activation and higher solution viscosity promoted the formation of larger domains and more pronounced vertical relief rather than a fine, regularly organized honeycomb pattern.

Figure 3 illustrates that thermal treatment modifies the HCP morphology. For the sample prepared with 2 wt.% PLLA on PTFE subjected to plasma treatment for 240 s, heating produced a coarser arrangement of large depressions separated by relatively broad polymer walls. Nevertheless, R_a_ remained almost unchanged, increasing only from 352 to 354 nm. This observation is important because it shows that similar average roughness values do not necessarily correspond to identical surface morphologies: thermal treatment can redistribute the polymer and alter pore geometry without substantially changing the overall height deviation. For the 240 s plasma-treated substrate coated with 3 wt.% PLLA, heating of plasma treated PTFE generated well-separated, rounded-to-polygonal depressions surrounded by narrow, strongly elevated boundaries, and roughness increased from 711 to 918 nm. In contrast, thermal treatment of the sample prepared with 3 wt.% PLLA after only 20 s of plasma exposure reduced R_a_ from 317 to 180 nm. Simultaneously, the morphology changed from one dominant large cavity with smaller satellite depressions to a denser population of smaller and more uniformly distributed pores. Overall, the AFM results show that PLLA concentration and plasma exposure time act synergistically in determining the dimensions and vertical development of the honeycomb structures. A 2 wt.% PLLA solution generally produces finer and more homogeneous patterns, particularly after 240 s of plasma treatment, whereas 3 wt.% PLLA favours larger cavities and substantially higher roughness. Consequently, R_a_ should be evaluated together with pore dimensions, homogeneity, and wall geometry when selecting the most suitable surface for biological applications, because these morphological features can collectively influence wettability, protein adsorption, and subsequent cellular interactions.

### 3.3. Scanning Electron Microscopy

**Figure 4:**
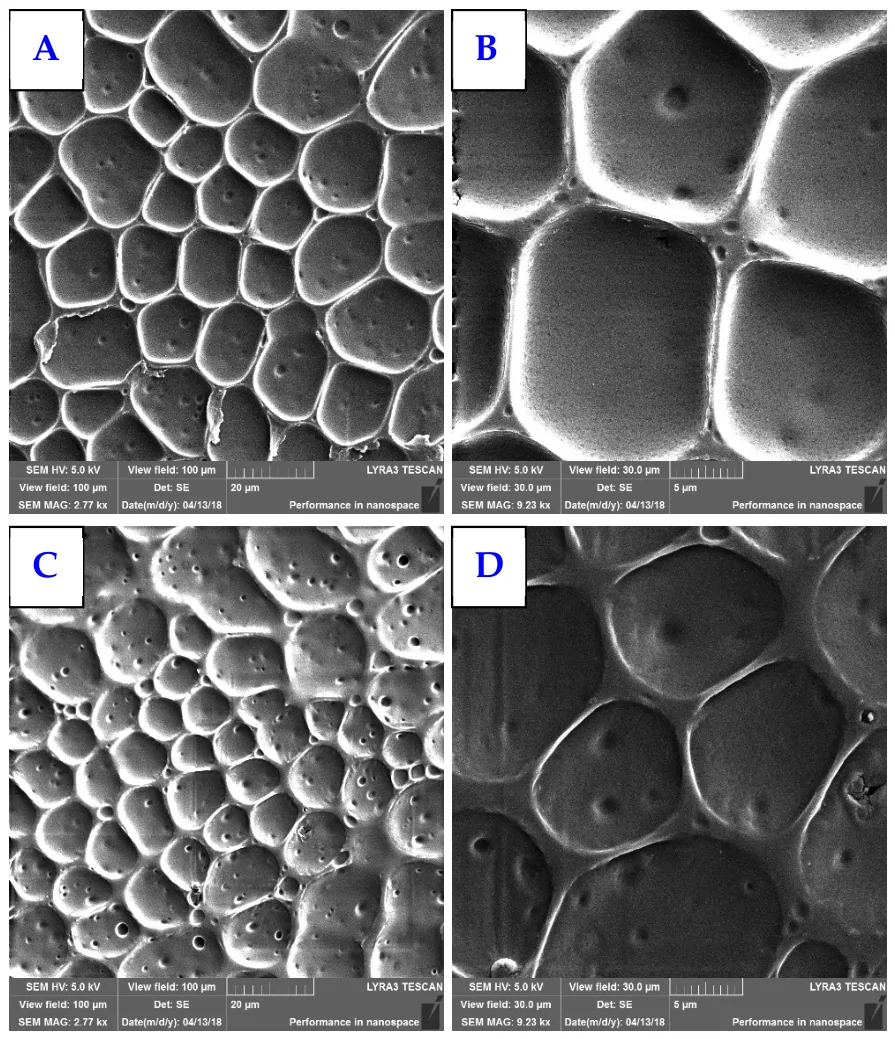
SEM scans of PLLA on PTFE substrate ((**A**) 100 micron scan and (**B**) 30 micron scan; 8 W and 20 s exposure, no heat treatment, and PLLA 2 wt. %), and SEM scans of PLLA on PTFE substrate ((**C**) 100 micron scan and (**D**) 30 micron scan; 8 W and 20 s exposure, no heat treatment, PLLA 3 wt. %).

**Figure 5:**
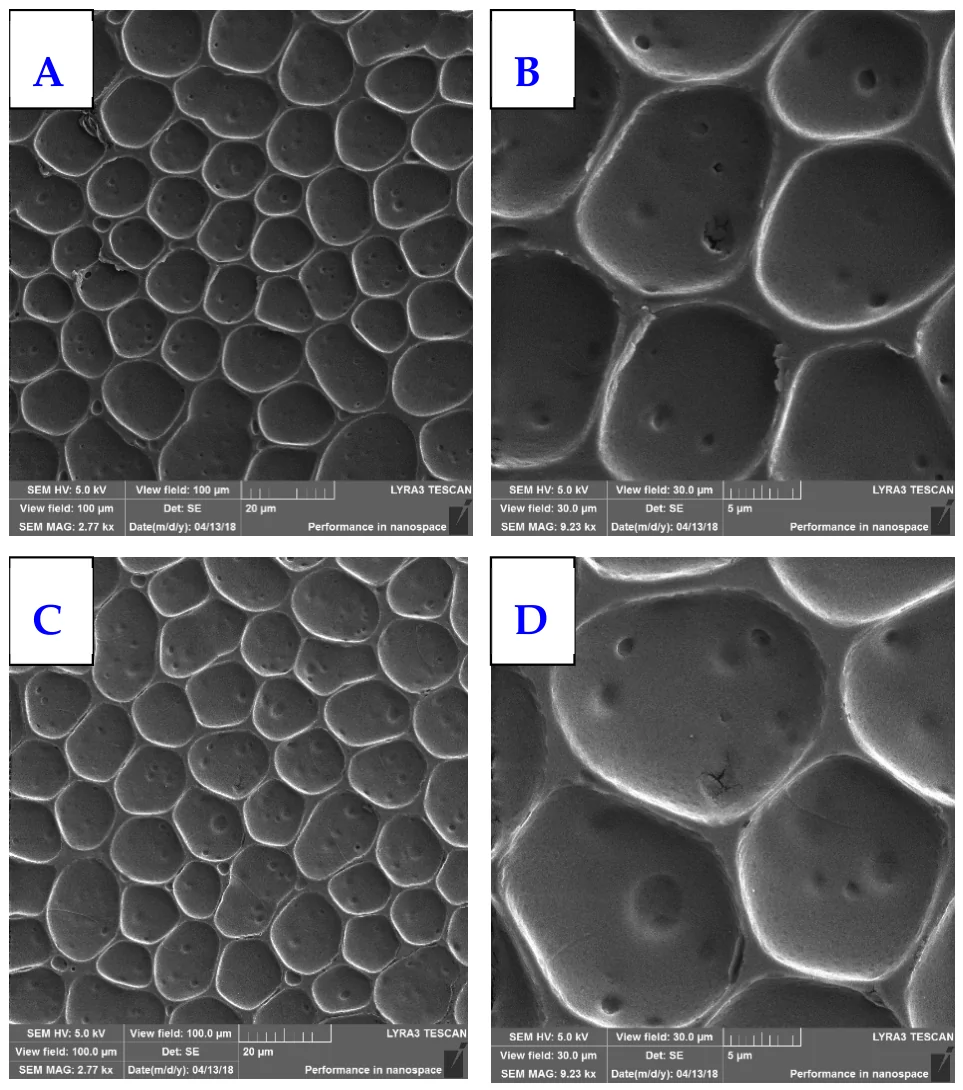
SEM scans of PLLA on PTFE substrate ((**A**) 100 micron scan and (**B**) 30 micron scan; 8 W and 240 s exposure, no heat treatment, and PLLA 2 wt. %) and SEM scans of PLLA on PTFE substrate ((**C**) 100 micron scan and (**D**) 30 micron scan; 8 W and 240 s exposure, no heat treatment, and PLLA 3 wt. %).

**Figure 6:**
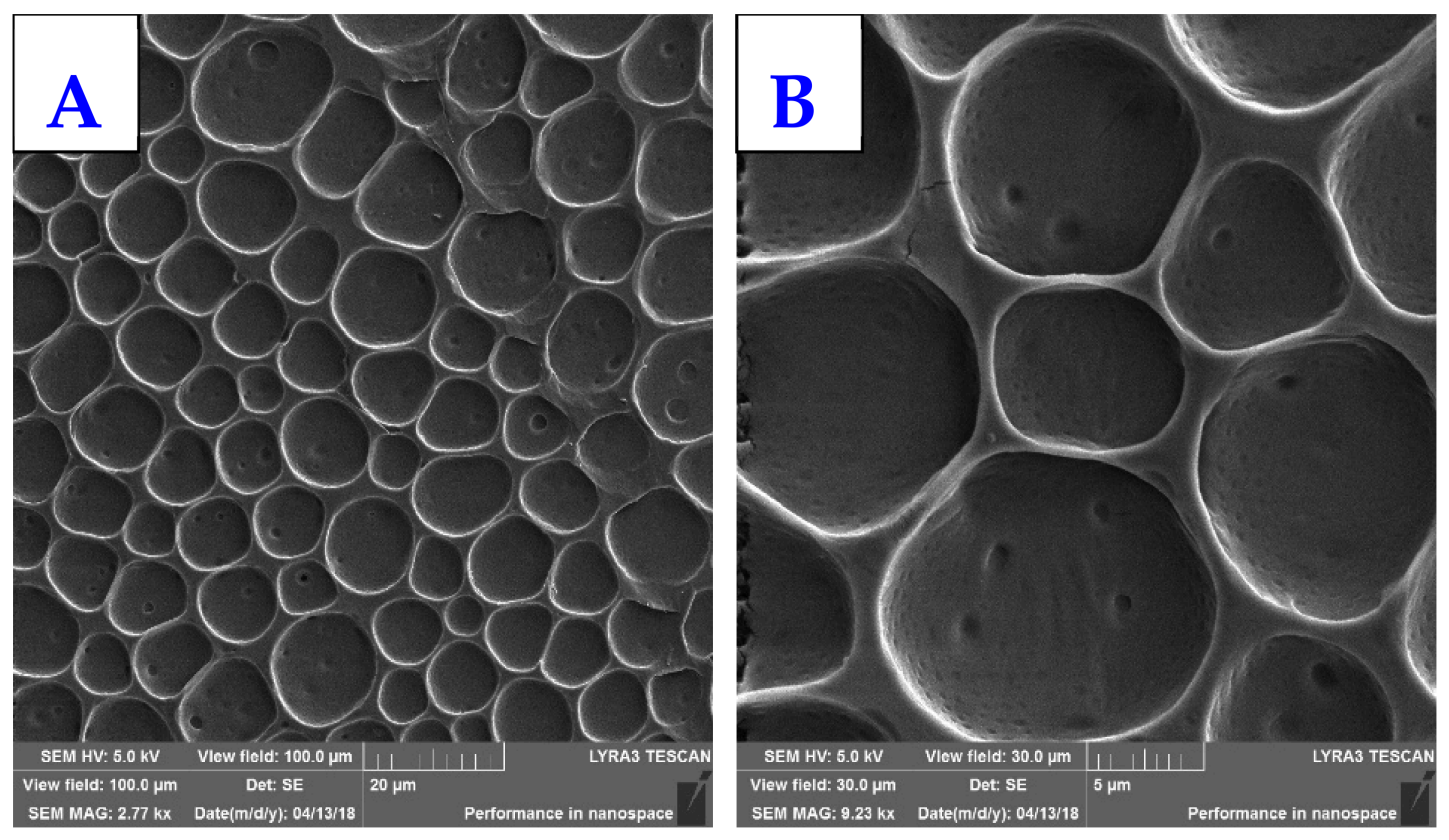
SEM scans of PLLA on PTFE substrate ((**A**) 100 micron scan and (**B**) 30 micron scan; 8 W and 240 s exposure, with heat treatment of PTFE after exposure 300 °C 1 h, PLLA 3 wt. %).

The SEM micrographs presented in Figure 4, Figure 5 and Figure 6 complement the AFM results by revealing the lateral organization, homogeneity, and continuity of the PLLA honeycomb-patterned structures over substantially larger surface areas. In all investigated samples, the deposited PLLA layer consists of closely packed, rounded-to-polygonal depressions separated by brighter, elevated polymer boundaries. The enhanced brightness of these boundaries is consistent with secondary-electron edge contrast and supports the presence of raised walls around the individual pore-like domains. The SEM observations therefore confirm that the PLLA patterns are not isolated local features but extend across relatively broad areas of the plasma-modified PTFE substrates. Figure 4 shows the structures formed after a short plasma exposure of 20 s. At a PLLA concentration of 2 wt.% (Figure 4A,B), the surface exhibits a compact network of rounded and polygonal domains with lateral dimensions generally within the micrometre to tens-of-micrometres range. The depressions are separated by continuous and clearly defined polymer walls, although some variation in their size and shape is visible. The higher-magnification image in Figure 4B demonstrates relatively smooth interiors and well-developed peripheral boundaries. Only a limited number of small secondary depressions or pinhole-like defects are observed within the larger domains. These findings correspond well with the comparatively low AFM roughness of Ra = 208 nm determined for this sample.

Increasing the PLLA concentration to 3 wt.% while maintaining the 20 s plasma exposure produces a more heterogeneous morphology (Figure 4C,D). The pattern remains continuous, but the distribution of domain dimensions becomes broader, and some structures appear larger and less regularly shaped. Moreover, a higher population of small secondary depressions is visible, particularly in the lower-magnification image. This morphology suggests that the increased viscosity and thickness of the deposited polymer solution affected the phase-separation process and permitted local growth or partial coalescence of the phase-separated domains. The more pronounced heterogeneity agrees with the increase in R_a_ from 208 to 317 nm observed by AFM. The effect of prolonged plasma treatment is demonstrated in Figure 5. After 240 s of plasma exposure, the 2 wt.% PLLA sample exhibits a dense and comparatively homogeneous arrangement of rounded-to-polygonal depressions (Figure 5A,B). The boundaries are generally continuous, and extensive cracking or delamination of the polymer layer is not observed. Compared with the corresponding 20 s sample, the pattern appears more uniformly distributed across the broader field of view. This improvement may be associated with more extensive activation of the PTFE surface, leading to more favourable spreading and stabilization of the PLLA solution during pattern formation. Small isolated pits remain present inside some domains, but these features do not significantly disrupt the overall structure.

On the PTFE samples with 3 wt.% PLLA and 240 s of plasma exposure (Figure 5C,D), the HCP morphology is also well developed and covers the substrate continuously. Individual domains are generally larger and exhibit a somewhat broader size distribution than those formed from the 2 wt.% solution. The high-magnification image reveals well-defined boundaries together with occasional secondary depressions and localized imperfections. Although the lateral morphology observed by SEM remains relatively regular, AFM detected a pronounced increase in R_a_ to 711 nm. This apparent difference highlights the complementary character of the two techniques: plan-view SEM primarily reveals lateral organization, whereas AFM provides quantitative information about vertical relief. Consequently, samples with comparable lateral pore dimensions may possess markedly different wall heights or depression depths. Figure 6 demonstrates the additional influence of heating the plasma-modified PTFE substrate at 300 °C for 1 h before applying the 3 wt.% PLLA layer. The resulting surface retains a continuous honeycomb-like morphology, but its size distribution appears more heterogeneous, with smaller domains occupying spaces between larger structures. The higher-magnification image shows rounded depressions surrounded by distinct and comparatively broad boundaries, while the interiors remain relatively smooth apart from several small pinhole-like features. No extensive failure of the PLLA layer is evident. Since the thermal treatment was applied to PTFE after plasma activation, it probably altered the physicochemical state of the substrate, including the stability or orientation of plasma-generated surface functionalities and, potentially, the surface morphology. These changes could modify the wettability and interfacial interaction of PTFE with the PLLA solution, thereby influencing its spreading and subsequent phase separation. The stronger topographic development is consistent with the increase in AFM roughness from 711 to 918 nm. An exploratory plan-view analysis was performed on surface depressions of SEM fields. Projected depression areas were converted to equivalent circular diameters using the instrument-reported horizontal field width. The selected depressions had equivalent diameters of approximately 9–22 µm. These measurements provide an estimate of the feature size scale within the introduced regions. The pore dimensions for particular samples, equivalent diameter, and mean ± SD (detected from SEM images) for the samples are as follows: 20 s plasma, 2 wt.% PLLA (15.8 ± 3.9) µm; 20 s plasma, 3 wt.% PLLA (10.1 ± 0.7) µm; 240 s plasma, 2 wt.% PLLA (14.0 ± 2.2) µm; 240 s plasma, 3 wt.% PLLA (14.9 ± 1.4) µm; and 240 s plasma, heated PTFE, 3 wt.% PLLA (12.3 ± 1.5) µm.

Overall, the SEM observations confirm that plasma-modified PTFE is a suitable substrate for forming continuous PLLA honeycomb structures. The 2 wt.% PLLA solution generally produces a finer and more homogeneous pattern, particularly following 240 s of plasma exposure, whereas the 3 wt.% solution promotes greater morphological heterogeneity and more pronounced vertical relief. Thermal treatment of plasma-modified PTFE further modifies the pattern without destroying its overall integrity. The combined SEM and AFM findings therefore demonstrate that plasma duration, PLLA concentration, and substrate heat treatment can be used to regulate both the lateral dimensions and vertical topography of the prepared HCP surfaces. Selected detailed SEM images are introduced in Figure S1.

### 3.4. X-Ray Photoelectron Spectroscopy

The chemical composition of PTFE polymer substrates subjected to plasma modification with a plasma power of 8 W, thermal stress (300 °C, 1 h), and subsequent phase separation, which led to the formation of HCP structures, was analysed using the X-ray photoelectron spectroscopy method. The following table (Table 2) summarizes the results of the chemical composition of selected PTFE substrates determined using the XPS method.

**Figure 7:**
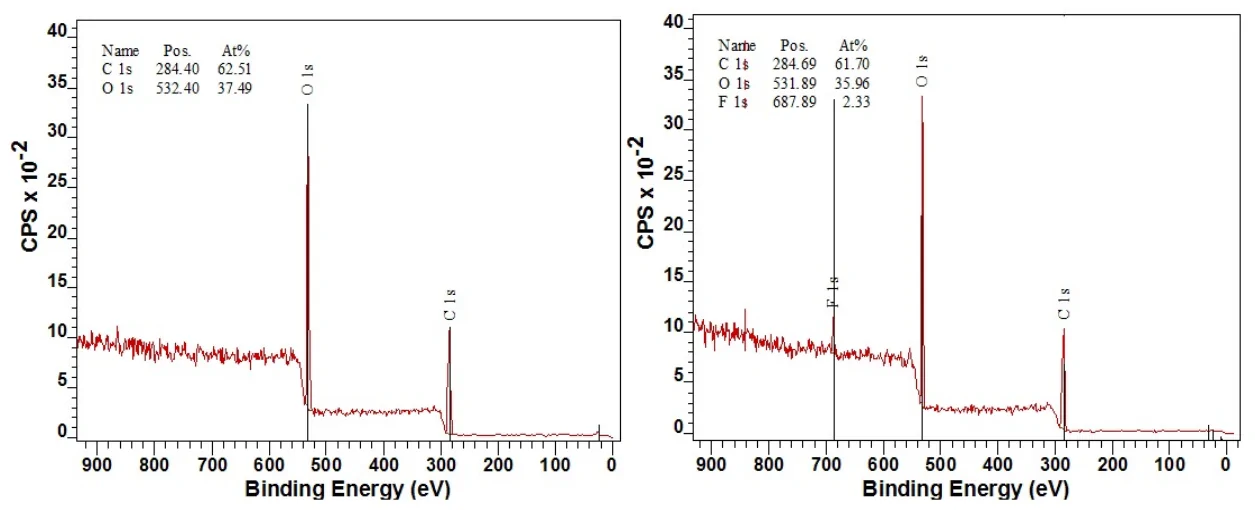
XPS spectrum of PTFE modified with Ar plasma 8 W for 20 s and 240 s and coated with a solution of 2 wt.% PLLA. Below, we present a table with the chemical composition of selected PTFE substrates analysed by XPS, with corresponding atomic concentrations of carbon, oxygen and fluorine.

The results in Table 2 show that plasma modification, thermal stress, and phase separation lead to changes in the quantitative composition on the surface of PTFE substrates by the formation of HCP structures and their modification. In unmodified PTFE, F is significantly represented (approx. 66.5%), C is present to a lesser extent (approx. 33.4%), and O is present in a minimal amount (approx. 0.1%). Plasma modification and phase separation lead to an increase in the amount of carbon and oxygen on the polymer surface, and the fluorine content is suppressed to a minimum (the maximum amount corresponds to 2.3% for a sample modified for 240 s and coated in a solution with 2 wt. % PLLA), which corresponds to the formation of a PLLA layer on the PTFE surface. In samples that were exposed to thermal stress, the fluorine content was equal to 0%, which represents a continuous PLLA layer. From these results (especially from the minimal F content on the surface of the samples), it can be concluded that plasma modification, thermal stress, and phase separation lead to almost 100% coverage of the surface of PTFE substrates with a PLLA layer and an increase in the surface amount of O (due to the oxidative effects of the plasma used during modification and especially the presence of PLLA) and a significant increase in the surface amount of C (PLLA layer). The following images show the spectra of two selected PTFE substrates taken using the XPS method (Figure 7).

### 3.5. Surface Wettability

After modification of PTFE substrates with argon plasma with a power of 8 W, subsequent thermal stress and phase separation, the samples were studied using goniometry. The measuring liquid was water and glycerol. Depending on the exposure time in the plasma, thermal stress and the PLLA content in the coating solution, changes in the wetting contact angles were observed. The following table (Table 3) summarizes the values of the wetting contact angles measured using goniometric analysis using a sessile drop of the measuring liquid (water/glycerol) on selected PTFE samples. Upon comparing the measured values of the modified PTFE samples with HCP structures with the contact angle values of the unmodified PTFE substrate, it is evident that the hydrophilicity (wetting ability) of the polymer surface increased, as the contact angle values decreased.

**Figure 8:**
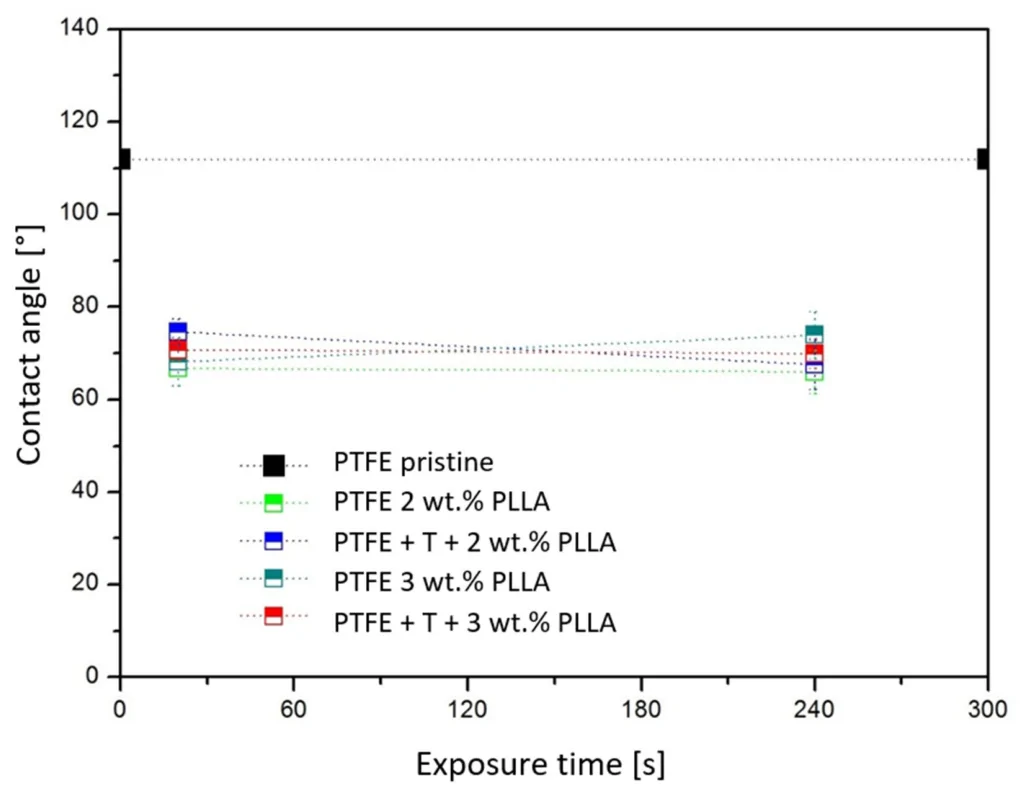
Dependence of the contact angle of PLLA on PTFE substrates on the modification time (20 s and 240 s), thermal stress, and PLLA content in the solution (2 wt.% and 3 wt.%).

The decrease in the contact angles on the modified samples can be explained by the increased surface amount of oxygen (as demonstrated by the XPS method). The largest decrease in contact angle compared to unmodified PTFE occurred for the sample modified for 240 s without thermal stress and coated in a solution with 3 wt.% PLLA, when the measuring liquid was glycerol. The contact angle values for the samples after plasma modification and phase separation and for both measuring liquids generally ranged from 60° to approximately 80°, which corresponds to the values characterizing hydrophilic materials; a graphical representation of values is shown in Figure 8.

### 3.6. Cytocompatibility

**Figure 9:**
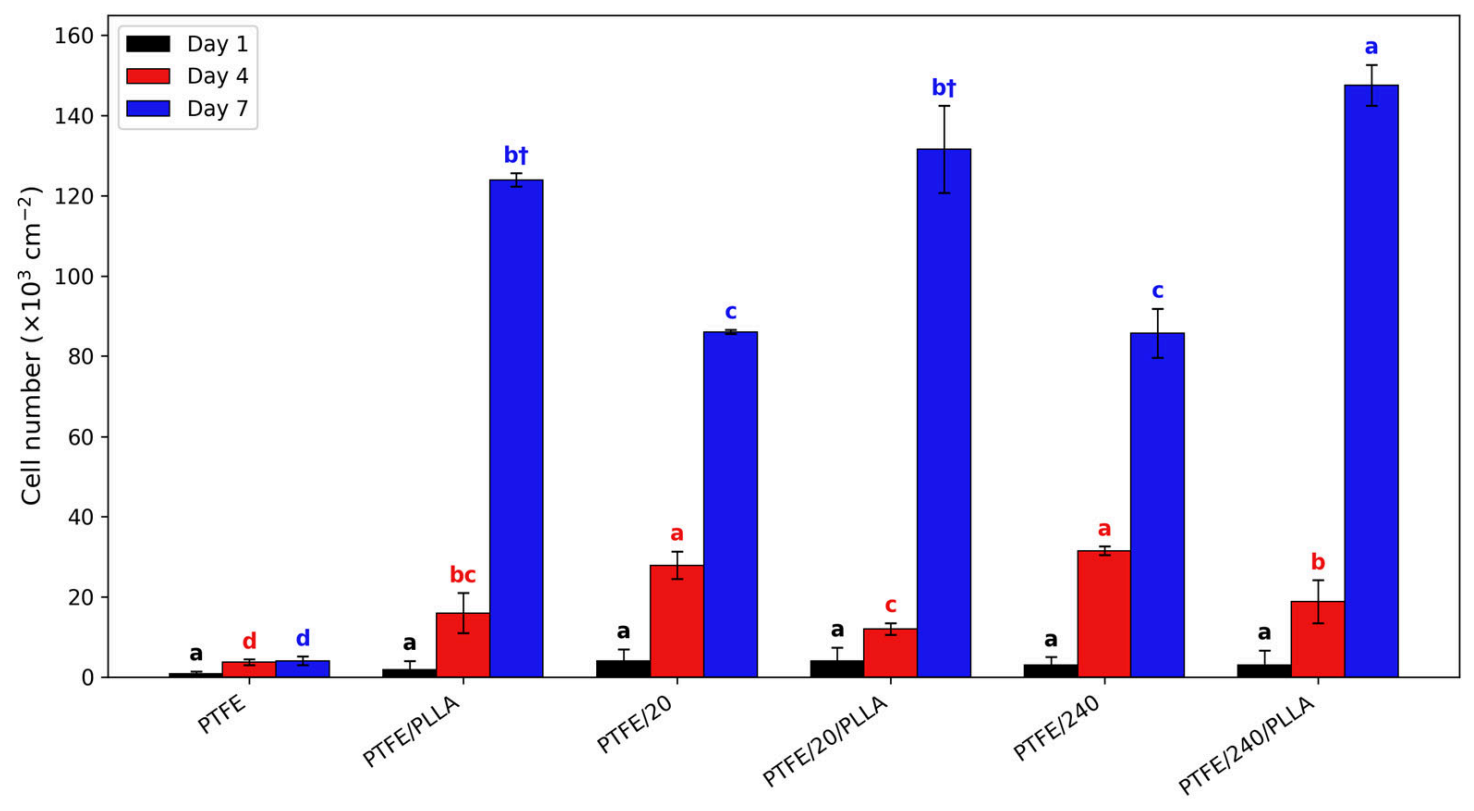
Dependence of cell number on plasma exposure time and presence of HCP PLLA structures on PTFE substrates. The results are shown for days 1, 4, and 7 after seeding. ANOVA + Tukey (HSD) analysis of the differences between the categories with a confidence interval of 95% (*n* = 4 independent replicates). Letters above the bars indicate Tukey HSD groupings at α = 0.05: within the same day, bars sharing at least one letter do not differ significantly, whereas bars with no letter in common differ significantly; thus, “bc” overlaps with both “b” and “c.” The dagger (†) identifies the day-7 comparison sensitive to image-reading precision.

The interaction of the polymer surface with tissue cultures was studied by in vitro methods. On selected PTFE samples (plasma power 8 W, exposure time 20 s and 240 s, without thermal stress, solvent ratio CHCl_3_/CH_3_OH equal to 90/10, 3 wt.% PLLA content in the solution) SaOS-2 cells (human osteoblasts) of the fifth passage were cultured. Before all biological tests, the samples were sterilized for 1 h in 75% ethanol. Briefly, 17,000 cells.cm^−2^ were seeded on each sample. Cultivation was carried out for 24, 96, and 168 h at a temperature of 37 °C, humidity 85%, in an air atmosphere with 5% CO_2_. The following image (Figure 9) shows the dependence of the number of cells cultured on PTFE substrates on plasma modification, plasma exposure time, and the presence of HCP PLLA microstructures on these substrates.

**Figure 10:**
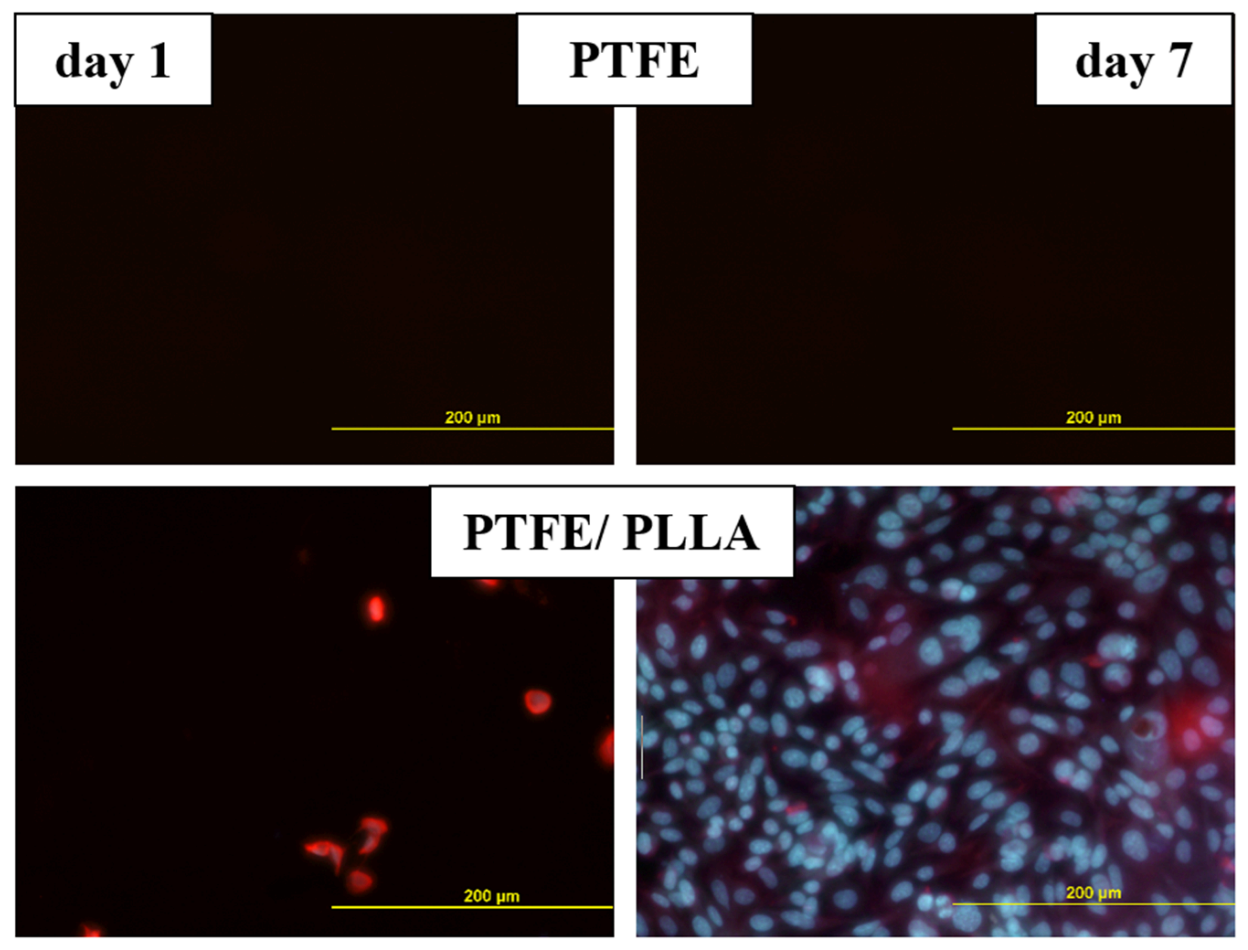
Biocompatibility testing of PTFE (pristine) and PTFE with 3 wt. % PLLA substrates. Results are from the 1st and 7th day of culture.

**Figure 11:**
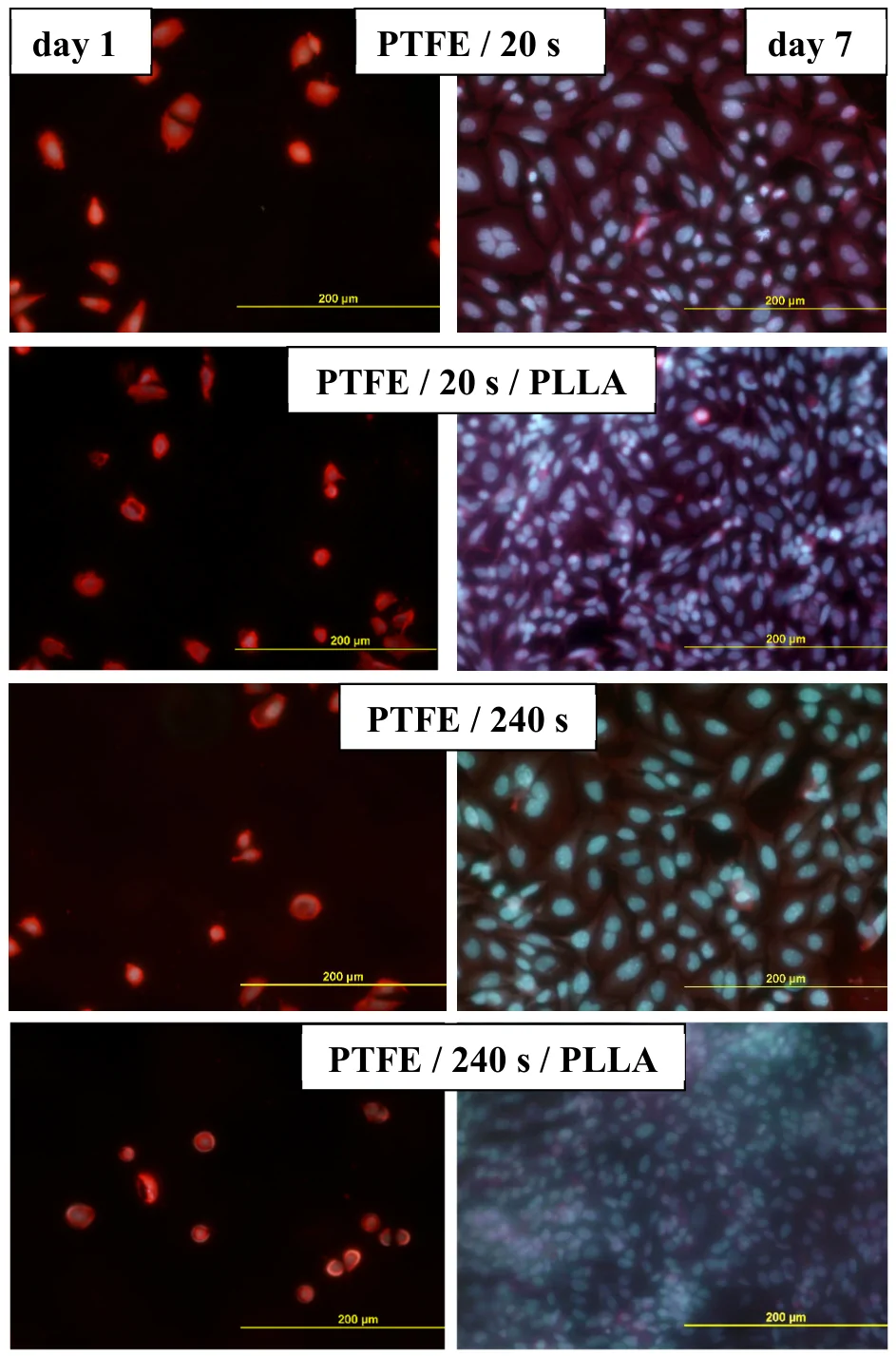
Biocompatibility testing of plasma-modified PTFE substrates without a PLLA layer and with 3 wt. % PLLA. Results are from the 1st and 7th day of cultivation.

From the values in Figure 9, it is evident that on the first day of cultivation, there was a slower proliferation of cells in the culture; the cells proliferated fastest on the PTFE substrate that was plasma-modified for 20 s and coated in a solution with 3 wt.% PLLA and the slowest on unmodified PTFE without PLLA. On the fourth day of cultivation, there was a large increase in the number of proliferated cells on the PTFE substrate after modification for 240 s in plasma without a PLLA layer; the lowest values of proliferated cells were again shown by the unmodified PTFE sample without PLLA. After the seventh day of cultivation, there was an exponential growth in the number of proliferated cells in all samples, except for the unmodified PTFE without PLLA. The most significant increase in proliferated cells was shown by the PTFE substrate modified for 240 s in plasma and with a layer of PLLA microstructures. The estimated number of proliferated cells on this sample was 150,000 cells per cm^−2^. Cell numbers increased descriptively between days 1 and 7 on all investigated surfaces, with the highest day-7 values observed on PLLA-coated PTFE. A one-way ANOVA reconstructed from the data means and 95% confidence intervals indicated no significant overall difference among surfaces on day 1 (*p* ≈ 0.079), whereas significant differences were estimated on days 4 and 7 (*p* < 0.001). On day 4, the plasma-treated surfaces without PLLA exhibited higher cell numbers than the PLLA-coated groups. By day 7, this relationship was reversed, and PTFE/240/PLLA showed the highest cell density, exceeding both PTFE/PLLA and PTFE/20/PLLA according to the estimated Tukey HSD comparisons. The difference between PTFE/PLLA and PTFE/20/PLLA was not significant in this reconstruction (*p* ≈ 0.061). Figure 10 and Figure 11 show the results of the biocompatibility of selected PTFE substrates after the 1st and 7th day of cultivation. When comparing the images of the unmodified PTFE substrate without a PLLA layer and the unmodified PTFE substrate with a PLLA layer (Figure 10), a strong difference can be noted not only in the adhesion of SaOS-2 cells to the given substrates, but also in their proliferation.

Direct comparison of the PLLA-coated groups showed that substantial SaOS-2 cell accumulation occurred even without plasma pre-treatment. At day 7, the observed cell density increased from approximately 1.24 × 10^5^ cells cm^−2^ on PLLA-coated PTFE without plasma pre-treatment to approximately 1.32 × 10^5^ and 1.49 × 10^5^ cells cm^−2^ after plasma exposure for 20 and 240 s, respectively. These values represent additional increases of approximately 6% and 19%. Thus, although PLLA coating was associated with the largest improvement relative to pristine PTFE, plasma pre-treatment was associated with a smaller additional increase at day 7, being most apparent after 240 s. This pattern was not consistent across the earlier endpoints and is interpreted descriptively rather than as evidence of established enhancement of proliferation.

The relative cell densities on plasma-treated PTFE and PLLA-coated plasma-treated PTFE changed during cultivation: the uncoated plasma-treated surface supported higher cell density on day 4, whereas the PLLA-coated surface supported higher density on day 7. This temporal reversal suggests that surface properties influence different stages of cell colonization. Plasma-induced changes in PTFE surface chemistry and morphology may provide a favourable interface for early post-attachment growth. Following PLLA deposition, however, the cell-contacting interface is governed primarily by the coating chemistry and honeycomb morphology; the plasma pre-treatment also influences the formation of this coating. The comparable initial attachment on the coated and uncoated plasma-treated substrates indicates that the subsequent differences cannot be explained solely by initial adhesion efficiency. The greater cell spreading observed on uncoated plasma-treated PTFE suggests different spatial interactions with the two interfaces.

On the honeycomb coating, elevated walls and depressions may alter the distribution of adhesive contacts and require additional cytoskeletal adaptation before sustained population expansion. At later times, a cell-derived extracellular matrix may progressively modify the local adhesive environment and facilitate continued colonization of the patterned surface. This provides a plausible explanation for the higher cell density on PLLA-coated PTFE on day 7, although these processes were not directly examined. Because PLLA deposition changes both surface chemistry and topography, their individual contributions cannot be separated using the present experimental design. Moreover, the measured cell densities represent net cell accumulation and do not independently establish changes in division rate, survival, or osteogenic differentiation.

In the case of the unmodified PTFE without a PLLA layer, the adhesion was almost zero, and even after 7 days of cultivation, there was no significant proliferation of cells on this substrate. In the case of the unmodified PTFE with a PLLA layer, not only did the cells adhere to this substrate; there was also an increase in the proliferation of cell cultures after 7 days of cultivation (for the approximate number of cells, see Figure 9). From Figure 11, it is evident that in plasma-modified PTFE samples without a PLLA layer and with a PLLA layer, cell adhesion was comparable. The difference was mainly in the subsequent proliferation, when on substrates with a PLLA layer, there was a more significant cell multiplication than on substrates without a PLLA layer. In plasma-modified PTFE samples without a PLLA layer, there was a greater spread of cells (i.e., the cells had a larger surface area). Cells cultured on the tested PTFE substrates had a typical shape and size and homogeneously covered the entire surface of the samples.

## 4. Conclusions

This study demonstrates the successful development of honeycomb PLLA microstructures on plasma-modified polymer PTFE using the improved phase separation method. Laser confocal microscopy (LCM), atomic force microscopy (AFM), and scanning electron microscopy (SEM) were used to detect changes in surface morphology and characterize the size and shape of the prepared PLLA microstructures. X-ray photoelectron spectroscopy (XPS) was used to determine the chemical composition of the surface of modified substrates with the formed HCP PLLA microstructures. The changes and properties of polymer substrates subjected to argon plasma modification, thermal stress, and subsequent phase separation using a mixture of chloroform and methanol solvents in a ratio of 90/10 with a PLLA content of 2 wt.% or 3 wt.% were determined using goniometric measurements, when the contact angle of the wetting of a sessile drop of the measuring liquid (water or glycerol) was measured in order to determine the hydrophilicity/hydrophobicity of the substrate surface. Atomic force microscopy (AFM) and scanning electron microscopy (SEM) confirmed the formation of PLLA microstructures on plasma exposer PTFE and that the size of these microstructures is influenced not only by the exposure time of the samples in plasma but also by the PLLA content in the coating solution and thermal stress of the plasma-exposed substrates. The chemical composition of the surface of PTFE substrates with the formed HCP microstructures was analysed by X-ray photoelectron spectroscopy (XPS). The results show that plasma modification and subsequent phase separation significantly increase the surface amount of oxygen and carbon and suppress the surface amount of fluorine to a minimum, which is evidence of almost 100% coverage of the PTFE surface with the coating solution with PLLA. We found that plasma modification and phase separation lead to a significant decrease in contact angle values or an increase in the hydrophilicity of the surface (wetting). Biocompatibility testing on selected PTFE samples revealed that modification with 8 W plasma in combination with HCP microstructures created by the phase separation method in solution with 3 wt.% PLLA supports cell culture proliferation in vitro.

## Figures and Tables

**Table 1:** Substrates evaluated by LCM as materials suitable for further analysis and biocompatibility testing.

PTFE Substrate, Plasma Power 8 W, Solvent Ratio CHCl_3_/CH_3_OH Equal to 90/10	HCP Appearance	Suitable for Next Analysis
Pristine PTFE + 2 wt.% PLLA	yes	no
Pristine PTFE + ΔT + 2 wt.% PLLA	yes	no
Pristine PTFE + 3 wt.% PLLA	yes	yes
Pristine PTFE + ΔT + 3 wt.% PLLA	yes	no
PTFE 20 s + 2 wt.% PLLA	yes	yes
PTFE 20 s + ΔT + 2 wt.% PLLA	yes	no
PTFE 20 s + 3 wt.% PLLA	yes	yes
PTFE 20 s + ΔT + 3 wt.% PLLA	yes	no
PTFE 240 s + 2 wt.% PLLA	yes	yes
PTFE 240 s + ΔT + 2 wt.% PLLA	yes	no
PTFE 240 s + 3 wt.% PLLA	yes	yes
PTFE 240 s + ΔT + 3 wt.% PLLA	yes	yes

**Table 2:** Chemical composition of selected PTFE substrates analysed by XPS method.

	C [%]	O [%]	F [%]
Pristine PTFE	33.4	0.1	66.5
Pristine PTFE + 3 wt.% PLLA	65.0	35.0	0
Pristine PTFE + ΔT + 3 wt.% PLLA	61.2	38.8	0
PTFE 20 s + 2 wt. % PLLA	62.5	37.5	0
PTFE 20 s + 3 wt.% PLLA	62.9	35.2	1.9
PTFE 20 s + ΔT + 3 wt.% PLLA	63.3	36.7	0
PTFE 240 s + 2 wt.% PLLA	61.7	36.0	2.3
PTFE 240 s + 3 wt.% PLLA	63.5	34.7	1.8
PTFE 240 s + ΔT + 3 wt.% PLLA	63.3	36.7	0

**Table 3:** Values of measured contact angles of selected PTFE samples (plasma power 8 W).

	Contact Angle (Water) [°]	Contact Angle (Glycerol) [°]
Pristine PTFE	111.9 ± 1.6	103.5 ± 2.6
PTFE 20 s	92.7 ± 5.6	93.5 ± 2.9
PTFE 240 s	83.5 ± 5.7	101.8 ± 2.4
Pristine PTFE + 2 wt.% PLLA	72.2 ± 4.3	68.1 ± 1.4
Pristine PTFE + ΔT + 2 wt.% PLLA	70.6 ± 3.3	65.6 ± 3.4
PTFE 20 s + 2 wt.% PLLA	66.7 ± 1.8	66.6 ± 2.4
PTFE 20 s + ΔT + 2 wt.% PLLA	74.7 ± 2.8	77.1 ± 4.1
PTFE 240 s + 2 wt.% PLLA	66.0 ± 4.7	71.4 ± 1.8
PTFE 240 s + ΔT + 2 wt.% PLLA	67.5 ± 5.3	75.3 ± 1.8
Pristine PTFE + 3 wt.% PLLA	72.7 ± 1.7	66.8 ± 3.1
Pristine PTFE+ ΔT + 3 wt.% PLLA	70.3 ± 0.4	72.1 ± 4.0
PTFE 20 s + 3 wt.% PLLA	68.1 ± 5.2	67.4 ± 2.6
PTFE 20 s+ ΔT + 3 wt.% PLLA	70.7 ± 2.1	68.3 ± 1.3
PTFE 240 s + 3 wt.% PLLA	73.9 ± 5.2	61.9 ± 3.1
PTFE 240 s + ΔT + 3 wt.% PLLA	69.9 ± 3.2	73.6 ± 3.4

## Data Availability

The data presented in this study are available at https://doi.org/10.5281/zenodo.23694756.

## Supplementary Materials

The following supporting information can be downloaded at: https://www.mdpi.com/article/10.3390/polym18212800/s1, Figure S1: Detailed SEM scans of PLLA on PTFE substrates.
