# Supplementary material for: A Poly-L-Lactic Acid Honeycomb Scaffold for Enhanced Cytocompatibility
Source: Polymers (Basel). 2026 Nov 9;18(21):2800. doi: 10.3390/polym18212800 (PMC13766037; doi:10.3390/polym18212800)
Supplement: Supplementary file 1 [file polymers-18-02800-s001.zip › polymers-4607913-supplementary.pdf]

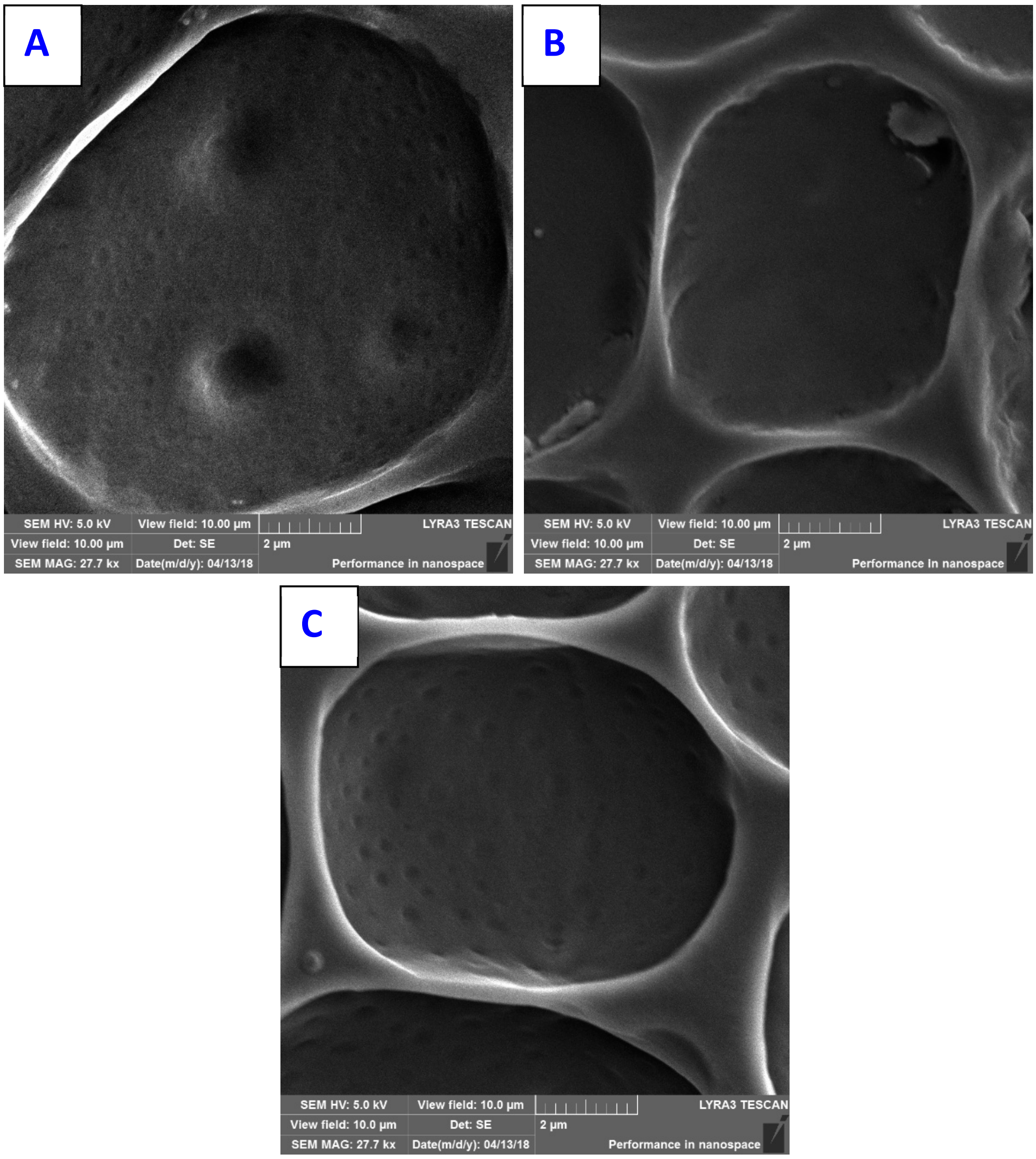

Figure S1 Detailed SEM scans of PLLA on PTFE substrate with no heat treatment, 10 micron scans, A - 8 W and 20 s + PLLA 3 wt. %, B - 8 W and 240 s + PLLA 2 wt. %; and with heat treatment, 10 micron scan - C - 8 W and 240 s + PLLA 3 wt. %.
